# The effect of cytokines on osteoblasts and osteoclasts in bone remodeling in osteoporosis: a review

**DOI:** 10.3389/fimmu.2023.1222129

**Published:** 2023-07-05

**Authors:** Jie Xu, Linxin Yu, Feng Liu, Longbiao Wan, Zhenhua Deng

**Affiliations:** ^1^ Department of Orthopedics, Renmin Hospital of Wuhan University, Wuhan, China; ^2^ Renmin Hospital of Wuhan University, Wuhan, China; ^3^ Hubei Provincial Hospital of Traditional Chinese Medicine (TCM), Wuhan, China

**Keywords:** osteoporosis, cytokine, osteoblasts, osteoclasts, bone remodeling

## Abstract

The complicated connections and cross talk between the skeletal system and the immune system are attracting more attention, which is developing into the field of Osteoimmunology. In this field, cytokines that are among osteoblasts and osteoclasts play a critical role in bone remodeling, which is a pathological process in the pathogenesis and development of osteoporosis. Those cytokines include the tumor necrosis factor (TNF) family, the interleukin (IL) family, interferon (IFN), chemokines, and so on, most of which influence the bone microenvironment, osteoblasts, and osteoclasts. This review summarizes the effect of cytokines on osteoblasts and osteoclasts in bone remodeling in osteoporosis, aiming to providing the latest reference to the role of immunology in osteoporosis.

## Introduction

Osteoporosis is an orthopedic disease characterized by bone mass reduction and bone tissue microstructure damage, which can increase bone fragility and the incidence of fractures (1). Osteoporosis can occur at any age, but is more common in postmenopausal women and in elderly men, indicating a close association with aging (2). Given the health impact of osteoporosis for the increasingly middle-aged and elderly global population, osteoporosis has become a chronic disease that causes a huge disease burden and great socioeconomic pressure (3, 4).

In the normal body microenvironment, there is a balance between bone formation by osteoblasts and bone resorption by osteoclasts, supporting the bone mass and bone mineral density (BMD) within the normal range. However, this balance is disrupted in osteoporosis patients and the bone resorption exceeds bone formation, initiated by the suppression of osteoblasts or the over-activation of osteoclasts, which is referred to as bone remodeling (5). In this pathological process, the immune system plays a significant role, including through immune cells, various cytokines, and signaling pathways. The cross talk between the skeletal system and the immune system forms an interdisciplinary field called Osteoimmunology (6).

In the cytokine networks and signaling pathways, the receptor of NF-κB (RANK)/RANK ligand (RANKL)/osteoprotegerin (OPG) axis plays an important role in the bone remodeling process, which has already been researched systematically (7). Although the function of other proinflammatory cytokines, such as the tumor necrosis factor (TNF) family, the interleukin (IL) family, and interferon (IFN), has already been researched quite a lot in the bone remodeling literature (8), summary work remains in need. Therefore, in this review, we will describe the roles of various cytokines in bone remodeling and their impact on the pathological process of osteoporosis, combining recent discoveries (**Table 1**) in order to contribute to the search for new therapeutic targets for osteoporosis.

**Table 1:** Summary of the cytokine effects on osteoblasts and osteoclasts.

Cytokine	Effects on osteoblasts	Effects on osteoclasts	References
TNF-α	stimulate osteoblasts to express RANKL and M-CSF low concentration stimulates mesenchymal precursor cell differentiation into osteoblasts while high concentration inhibits osteoblasts’ function and bone formation inhibit IGF-1 and RUNX2 expression to suppress osteoblast differentiation	stimulate osteoclast differentiation promote RANK expression in osteoclast precursors promote RANKL-induced osteoclastogenesis induce osteoclast precursors to express c-Fos	(9–12, 13–15)
IL-1α	–	stimulate the formation of OLC	(16)
IL-1β	induce bone resorption in osteoblasts by activating p38 MAPK inhibit human osteoblast migration	activate osteoclasts and stimulate osteoclast differentiation, multinucleation, and survival	(17–20, 21, 22)
IL-3	increase osteoblast differentiation and matrix mineralization promote the expression of osteoblast-specific genes	inhibit RANKL-induced osteoclast differentiation inhibit TNF-induced osteoclast differentiation, bone resorption inhibit blood monocytes and bone marrow cells differentiate into osteoclasts	(23–29)
IL-4	–	directly and indirectly suppress osteoclastogenesis inhibit the bone resorption activity of mature, differentiated osteoclasts	(30–37)
IL-6	inhibit osteoblast differentiation	directly and indirectly stimulate osteoclast formation inhibit osteoclast progenitors to differentiate into osteoclasts	(38–43, 44, 45)
OSM	promote stromal cells to differentiate into osteoblast	stimulate RANKL production and osteoclast formation	(41, 46, 47)
IL-7	–	promote osteoclast formation by inducing T cells to produce RANKL and TNF-α promote bone resorption by inducing B cells increase stimulate osteoclast formation by activating STAT5	(48–50)
IL-8	–	promote RANKL-induced osteoclastogenesis	(51)
IL-10	inhibit bone marrow osteogenic activity	inhibit osteoclast progenitors differentiate into osteoclast precursors inhibit RANK-induced osteoclast formation	(52–56)
IL-11	extend the survival of osteoblast progenitor cells promote pluripotent progenitor cells to differentiate into osteoblast lineage promote osteogenesis, inhibit adipogenesis and sclerostin in osteoblasts	stimulate osteoclast differentiation and osteoclast formation	(38, 57–59, 46, 60)
IL-12	–	inhibit RANKL-induced osteoclastogenesis through inhibiting NFATc1 or promotion of osteoblast apoptosis *via* the Fas/FasL	(61, 62)
IL-13	–	inhibit osteoclast formation and bone resorption	(36, 63)
IL-15	promote osteoblast apoptosis	promote osteoclast progenitors to differentiate into osteoclast precursor induce osteoclast formation	(64–66)
IL-17	promote the expression of pro-osteoclastic cytokines such as TNF-α, IL-6, and RNAKL in osteoblasts promote osteoblast differentiation while inhibiting osteoblast calcification	induce osteoclastogenesis low concentrations promote autophagy of osteoclast precursors and osteoclast formation while high concentrations inhibit osteoclast precursors’ differentiation into osteoclasts	(67–79)
IL-18	–	inhibit TNF-α-induced osteoclastogenesis by mediating myeloid apoptosis via Fas/FasL and NO indirectly inhibit osteoclast formation via IFN-γ and GM-CSF	(80–84)
IL-19	–	inhibit RANKL-induced osteoclast differentiation maintain the osteoclast precursor state	(85)
IL-20	upregulate RANKL expression in osteoblasts inhibit osteoblasts survival and differentiation	induce the expression of RANK in M-CSF-derived osteoclast precursors and promote the transduction of osteoclastic signals	(86, 87)
IL-23	–	participate in T-cell-mediated osteoclast formation modulate osteoclast differentiation indirectly inhibit osteoclast formation	(88–91)
IL-27	inhibit osteoblast apoptosis	inhibit osteoclastogenesis	(92–95)
IL-29	–	inhibit osteoclast formation and bone resorption activity	(96, 97)
IL-32	promote bone formation and prevent bone loss	–	(98)
IL-33	stimulate osteoblast function promote matrix mineral deposition and reduces sclerostin mRNA	inhibit RANKL-induced osteoclast formation and osteoblast-related gene expression induce osteoclasts apoptosis inhibit TNF-induced osteoclast formation and bone resorption	(99–101, 102, 103)
IL-34	regulate hBMSC osteogenesis and enhance fracture healing	induce osteoclast differentiation and bone resorption promote the proliferation and differentiation of BMMs toward osteoclasts	(104–106)
IL-35	stimulate MSCs to differentiate into osteoblasts	prevent TNF-induced osteoclast formation and promote apoptosis promote functional osteoclast formation increase osteoclast differentiation factors expression	(107–109)
IL-37	increase the expression of osteoblast-specific genes promote osteogenic differentiation of MSCs	inhibit osteoclast formation and pathological bone resorption	(110, 111)
IFN-α	–	inhibit RANKL-induced osteoclastogenesis by reducing c-Fos	(112, 113)
IFN-β	–	inhibit RANKL-induced osteoclastogenesis by reducing c-Fos inhibit osteoclastogenesis by increasing NO production and the iNOS signaling pathway	(112–114)
IFN-γ	stimulate osteoblast differentiation genes expression stimulate osteoblast differentiation	inhibit osteoclast differentiation and function mediate osteoclast apoptosis via Fas/FasL	(115–121)

## Molecular mechanism of osteoporosis

**Figure 1:**
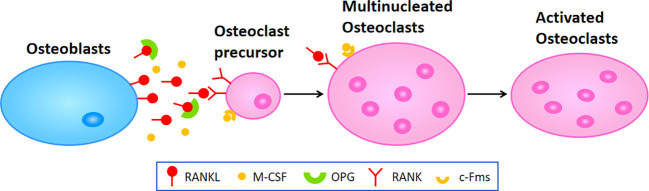
The molecular mechanism of osteoclastogenesis. Osteoblasts secret RANKL and M-CSF which respectively binding to RANK and c-Fms in osteoclast precursors or multinucleated osteoclasts to promote the differentiation or fusion during osteoclastogenesis, leading to the activation of osteoclasts in the end.

Osteoporosis happens when the balance between bone resorption by osteoclast cells and bone formation by osteoblast cells breaks down. In the process of osteoclast differentiation and activation, osteoblast cells express RANKL and OPG, participating in modulating the differentiation of osteoclast cells. RANKL binding to RANK and lead to the activation of osteoclast differentiation via activation of downstream signaling pathways, while OPG inhibit aforementioned effect through inhibition of RANKL-RANK interaction (122) (**Figure 1**). In addition, osteoblast cells secrete macrophage colony-stimulating factor (M-CSF) binding to colony-stimulating factor-1 receptor (c-Fms) in osteoclast cells, which leads to the activation of phosphoinositide 3-kinase (PI3K) and growth factor receptor bound protein 2 (Grb2) and further promotes Akt and ERK signaling in osteoclast precursors or mature osteoclast cells (8). It has been widely recognized that the RANK/RANKL/OPG axis plays a critical role in the molecular mechanism of osteoporosis. When pro-inflammatory cytokines affect this axis and enhance its osteoclastogenesis to a state of decompensation, bone mass reduces and the bone tissue microstructure becomes damaged, leading to the occurrence of osteoporosis.

## Tumor necrosis factor

TNF is subdivided into TNF-α and TNF-β, both of which strongly stimulate bone resorption. TNF contributes to the development of bone loss by fostering the formation of osteoclasts and inhibiting osteoblast function. TNF can stimulate osteoclast differentiation via multiple mechanisms, some of which are independent of the RANKL/RANK axis (123, 124). TNF-α stimulates stromal cells and osteoblasts and activates T cells to express the RANKL and M-CSF genes, which indirectly promote the expression of RANK in osteoclast precursors and subsequent osteoclastogenesis via M-CSF (9–11). In postmenopausal women with osteoporosis, TNF-α is closely associated with levels of RANK and estrogen. By activating NF-κB and PI3K/Akt signaling, TNF-α can promote RANKL-induced osteoclast formation *in vitro* in a synergistic manner (12). It may be one of the pathogenic mechanisms underlying osteoporosis after menopause.

Independent of the RANKL/RANK axis, TNF-α can directly exert biological effects. TNF-α induces the differentiation of osteoclast precursors by acting directly on their surface receptors and sequentially activating NF-κB, p50/p52, c-Fos, and nuclear factor-activated T cells c1 (NFATc1) (125, 126). TNF-α can also directly induce osteoclast precursors to express c-Fos, which produces IL-1β by interacting with bone matrix proteins and inducing osteoclast differentiation autocrine (127).

Tumor necrosis factor receptor-associated factors (TRAFs) are crucial in physiological bone remodeling and also influence TNF-induced osteoclast formation. TRAF2 (128) and TRAF5 (129) have been shown to be essential for TNF-α-induced osteoclast formation. The most important of the TRAFs is TRAF6, and mice lacking this factor develop severe osteoporosis due to inadequate osteoclast differentiation (130). However, as demonstrated in one study, RANKL might degrade the osteoclast formation inhibitor TRAF3 by inducing autophagic lysosomes, thereby stimulating TNF-induced osteoclast formation independent of TRAF6 signaling (131).

TNF has a concentration-dependent, bidirectional effect on bone formation and osteoblast function. Low concentrations of TNF stimulate mesenchymal precursor cells to differentiate into osteoblasts, whereas high concentrations of TNF inhibit osteoblast function and bone formation (13). TNF inhibits insulin-like growth factor I (IGF-I) expression in osteoblast precursor cells during the initial stages of differentiation, thereby inhibiting osteoblast differentiation (14). TNF also can inhibit the expression of RUNX2, a key factor regulating osteoblast differentiation, to suppress osteoblast differentiation (15).

## Interleukins

### Interleukin-1

Interleukin (IL)-1, composed of IL-1α and IL-1β, is an introductory class of cytokines that can activate osteoclasts and participate in osteoclast differentiation, multinucleation, and survival, which is also a crucial mediator of inflammatory bone loss (17). Among them, IL-1β is a decisive osteoclast factor that upregulates RANKL production and enhances its activity, and stimulates osteoclast formation (18, 19). IL-1β can indirectly promote TNF-α-induced osteoclastogenesis, induce stromal cell RANKL expression with the support of p38 MAPK, and thus directly stimulate osteoclast precursor differentiation (20). Interestingly, IL-1β has distinct effects on subpopulations of osteoclast precursors, stimulating polynucleation and bone resorption at varying rates in osteoclasts derived from the three precursors (132).

IL-1 is capable of RANKL/RANK-independent activation of osteoclast differentiation via IL-1/IL-1R1 signaling in bone marrow-derived macrophages (BMMs) by activating osteogenic marker genes such as NF-κB, JNK, p38, and ERK, as well as a microphthalmia transcription factor (MITF) to induce osteoclast differentiation. Nonetheless, this process does not stimulate c-Fos or NFATc1 expression and requires both to be at a certain baseline level (133). Not only do TNF and IL-1 induce bone resorption in osteoblasts by activating p38 MAPK in the osteoblast spectrum (21), but IL-1 also inhibits human osteoblast migration, thus impacting the fracture healing process (22).

In contrast, IL-1α can stimulate the formation of osteoclast-like cells (OLCs) by increasing the expression of M-CSF and PGE2 and decreasing the expression of OPG in osteoblasts (16).

### Interleukin-3

IL-3, also known as multicolony stimulation factor (multi-CSF), is a member of the βc family due to the fact that its receptor shares the signaling subunit βc with granulocyte/macrophage colony-stimulating factor (GM-CSF) and IL-5 (134). Activated T lymphocytes, mast cells, and osteoblasts are the primary secretors (135).

Although IL-3 is predominantly regarded as an anti-osteoclastic cytokine, it actually has a role in both osteoblasts and osteoclasts.

IL-3 inhibits RANKL-induced NF-κB nuclear translocation by inhibiting IκB phosphorylation and degradation, which further inhibits RANKL-induced osteoclast differentiation by acting directly on early osteoclast precursors (23). By downregulating the expression of TNFR1 and TNFR2, IL-3 inhibits TNF-induced osteoclast differentiation (24) and bone resorption (25). All of the above-mentioned IL-3 inhibition is irreversible. IL-3 substantially inhibits c-Fms, downregulating PU.1 and c-Fos expression at the mRNA and protein levels, leading to the suppression of differentiation of blood monocytes and bone marrow cells into osteoclasts and bone resorption, thereby inhibiting bone erosion (26–28).

In the osteogenesis process, IL-3 increases osteoblast differentiation and matrix mineralization in human mesenchymal stem cells (MSCs) in a dose-dependent manner, significantly boosting the expression of osteoblast-specific genes including alkaline phosphatase, collagen type-I, osteocalcin, and osteopontin, as well as the transcription factors Runx-2 and osterix (29). Through the JAK/STAT signaling pathway, IL-3 also promotes osteoblast differentiation and BMP-2 secretion (29).

Another study demonstrated that IL-3 increased RANKL expression at both the transcriptional and translational levels without affecting OPG expression. Increased RANKL induces mononuclear osteoblasts without affecting multinuclear osteoblasts. IL-3 regulated two functional forms of RANKL by downregulating metalloproteinases, including ADAM10, ADAM17, ADAM19, and MMP3, to downregulate soluble RANKL expression and increase membrane-bound RANKL expression via the JAK2/STAT5 signaling pathway, thereby restoring the decreased RANKL/OPG ratio in adult mice (136).

### Interleukin-4

IL-4 is a pleiotropic immunomodulatory cytokine produced primarily by Th2 cells, mast cells, and eosinophils (137). It regulates immune responses and has now been shown to be a potent inhibitor of osteoclastogenesis, affecting osteoclast formation and function in multiple ways, both directly and indirectly.

IL-4 reduces the nuclear translocation of NF-κB by inhibiting IκB phosphorylation in a STAT6-dependent manner, which significantly hinders the DNA-binding activity of NF-κB and directly suppresses osteoclastogenesis (30).

In addition, IL-4 can also inhibit RANKL and TNF-α-induced osteoclastogenesis by blocking the MAPK signaling pathway (31). Further studies showed that IL-4 inhibited the expression of NFATc1, a significant transcription factor for RANKL-induced osteoclast formation, *via* STAT6, thereby inhibiting osteoclast formation (32). In addition to inhibiting osteoclast formation, IL-4 directly inhibits the bone resorption activity of mature, differentiated osteoclasts by inhibiting the NF-κB and Ca^2+^ signaling pathways in a STAT6-dependent manner (33, 34). These are all direct inhibitory effects of IL-4 on osteoclastogenesis. By diminishing the production of pro-osteoclast factors such as TNF-α, IL-1, and IL-6, IL-4 also indirectly inhibits osteoclastogenesis (35). In contrast, endothelial cells of the bone vascular system secrete osteogenic cytokines and hormones to modulate bone development, remodeling, and repair. IL-4 and its closely related IL-13 can indirectly inhibit osteoclast formation by activating the STAT6 pathway to induce endothelial cells to secrete the osteoprotective hormone OPG (36).

IL-4 also has a synergistic effect with other cytokines, such as GM-CSF, in inhibiting osteoclastogenesis. In the presence of M-CSF and RANKL, monocytes differentiate into osteoclast. However in the presence of GM-CSF and IL-4, the combination of them upregulates TNF-a converting enzyme (TACE), causing M-CSF receptor shedding and monocytes are differentiating toward dendritic cells thereby disrupting osteoclastogenesis. (37).

## Interleukin-6 family

### Interleukin-6

The IL-6 family is a family of cytokines that share the signaling receptor subunit glycoprotein 130 kDa (gp130), including IL-6, IL-11, Oncostatin M (OSM), leukemia inhibitory factor (LIF), cardiotrophin 1 (CT-1), ciliary neurotrophic factor (CNTF), cardiotrophin-like cytokine factor 1 (CLCF1), neuropoietin (NP), IL-27, and humanin (138).

IL-6 is one of the pleiotropic cytokines that transmits signals by binding to the IL-6 receptor (IL-6R) (138). IL-6R is subdivided into the transmembrane receptor IL-6R and soluble IL-6R (sIL-6R), which exert biological effects via two distinct pathways including classic (cis) IL-6 signaling and alternative trans-signaling, respectively (139). IL-6 is produced by osteoblasts (140, 141), bone marrow stromal cells (BMSCs) (142), osteoclasts, macrophages (143), T cells (143), and neutrophils (144). Studies have indicated that IL-6 has been implicated in a number of age-related diseases, including osteoporosis, and its levels rise with age (145, 146), possibly through the P3IK/AKT, MAPK, and JAK/STAT pathways (147–149).

In the process of bone remodeling, IL-6 is primarily regarded as a pro-osteoclastic factor that promotes osteoclastic processes via direct and indirect pathways. Mouse models overexpressing IL-6 exhibit a phenotype characterized by an increase in osteoclasts and a reduction in bone trabecular volume (150). IL-6 directly stimulates osteoclast formation via a RANKL-independent mechanism, when OPG treatment does not inhibit this stimulatory effect (38), and therefore is regarded as a RANKL-independent stimulatory mechanism, although the precise mechanism was not revealed by the study. At the same time, IL-6 can indirectly stimulate osteoclastogenesis by stimulating RANKL produced by stromal and osteoblast cells (39–41) via JAK-mediated activation of STAT3 (42), resulting in an increase in the expression of osteoclast markers. Inhibition of IL-6R signaling inhibits osteoclast formation both *in vitro* and *in vivo* (151). A study showed that IL-6-deficient mice had increased bone mass, tartrate-resistant acid phosphatase (TRAP)-positive osteoclast numbers, and alkaline phosphatase activity in osteoblasts (152), indicating that IL-6 deficiency inhibited osteoclast maturation. In IL-6-deficient mice, osteoclast apoptosis was also increased (153). However, it has also been reported that IL-6 has a negative regulatory effect on osteoclast formation, and that IL-6 can act directly on osteoclast progenitors to inhibit their differentiation into osteoclasts by inhibiting the RANK signaling pathway-mediated degradation of IκB and activation of JNK (40). The differential regulation of osteoclasts by IL-6 may be attributable to the presence of varying concentrations of RANKL. IL-6/sIL-6R modulates NF-κB, ERK, and JNK signaling pathways differentially, inhibiting osteoclast formation at higher RANKL concentrations and promoting osteoclast formation at lower RANKL concentrations (43).

In addition, IL-6 contributes to the osteogenesis process. Compared with wild-type mice, IL-6 knockout mice after ovariectomy (OVX) demonstrated significant upregulation of mRNA for osteoblast-related genes, such as Runx2 and Col1a1, and downregulation of osteoclast-related genes, such as TRAP, MMP9, and CTSK (154). In a mouse model of osteoporosis, the inflammatory state of the microenvironment inhibits the osteogenic differentiation of BMSCs, and IL-6 is one of the most significant factors in this inhibition. The overactivated IL-6-STAT3 pathway inhibits β-catenin activity, and anti-IL-6 neutralizing antibodies rescue the osteoporotic phenotype in rodents (44), providing a potential therapeutic target for osteoporosis. In addition, the expression of TLR2, TLR4, IL-1, and TNF-α increases in BMSCs in response to IL-6, activating the AKT pathway and further inhibiting Setd7 expression. BMSCs, on the other hand, demonstrate a decrease in osteogenic gene expression and an increase in inflammatory gene expression (155). IL-6 inhibits osteoblast differentiation through activation of the JAK/STAT, SHP2/MEK2, and SHP2/AKT signaling pathways (45). In contrast to these findings, it has been hypothesized that the IL-6/IL-6R complex can activate the downstream STAT3 signaling pathway and promote osteogenic differentiation of bone marrow-derived mesenchymal stem cells (BM-MSCs) via an autocrine/paracrine feedback loop (156).

### Interleukin-11

IL-11, also known as adipogenesis inhibitory factor (AGIF) (157), is primarily secreted by stromal cells and osteoblasts (158) and shares the co-receptor gp130 with IL-6 family members (138). Therefore, IL-11 plays a similar function to IL-6 during bone remodeling. IL-11 can not only directly stimulate osteoclast differentiation independent of RANKL (38) but can also stimulate osteoclast formation indirectly through inducing RANKL production in osteoblastic lineage cells (57). The difference is that IL-11R is expressed in the osteoblast lineage (158) and IL-11 extends the survival of osteoblast progenitor cells (58). IL-11 has pro-osteogenic effects in addition to its pro-osteoclastic effects. *In vitro* administration of IL-11 promotes osteoblast lineage differentiation from pluripotent progenitor cells (59). The phenotype of mice overexpressing IL-11 was characterized by increased bone formation, thickened bone cortex thickness, and enhanced bone strength (159). In contrast, mice lacking IL-11R exhibited impaired bone formation on the trabecular surface and increased adipose in the bone marrow, indicating that IL-11R signaling is essential for osteoblast differentiation (160). Various studies have shown that IL-11 also promoted the process of osteogenesis when stimulated by mechanical loading. IL-11 is upregulated to promote osteogenesis, inhibit adipogenesis (60), and suppress the expression of sclerostin (an inhibitor of osteoblast differentiation, an osteoclast gene sensitive to mechanical stress) in osteoblasts (46). This process may be mediated through the Wnt signaling pathway (161, 162) and ΔFosB (161, 163).

### Oncostatin M

Oncostatin M (OSM), part of the IL-6 family, is expressed in all stages of osteoblast differentiation, including bone marrow stromal cells, stroma-producing osteoblasts, osteocytes, and bone-covered cells (46, 164). OSM expresses three receptor subunits, gp130, OSMR, and LIFR, in the osteoblast lineage, but not in osteoclasts (46). Additionally, bone macrophages can produce OSM (165–167) and bone morphogenetic proteins (BMPs) (168) to influence osteoblast differentiation. Depletion of bone macrophages can inhibit this differentiation process and is one of the mechanisms underlying diminished bone formation and bone growth in osteoporosis patients (169–171).

By stimulating RANKL transcription in stromal cells, OSM indirectly promotes osteoclast differentiation *in vitro* (41), but this effect is insufficient to completely support osteoclast formation *in vitro* (172). OSM can also promote the differentiation of stromal cells into osteoblasts rather than adipogenesis and inhibit sclerostin, an antagonist of Wnt pathway (46, 47). The two different effects mentioned above depend on the different receptors. OSM binds to gp130 first and forms a heterodimer with OSMR or LIFR, acting via LIFR to inhibit sclerostin production in stromal cell lines and osteoblasts, and acting via OSMR to stimulate RANKL production and osteoclast formation (46).

### Interleukin-7

IL-7 is a member of the IL-2 family and is primarily secreted by stromal cells and osteoblasts in response to the inflammatory cytokines IL-1 or TNF-α (11). Mice that overexpress IL-7 have a phenotype characterized by decreased bone mass and increased osteoclasts (173), suggesting that IL-7 also plays a role in bone remodeling. IL-7 can indirectly promote osteoclast formation by inducing T lymphocytes to produce RANKL and TNF-α (48), whereas in nude mice lacking T lymphocytes, IL-7 failed to cause bone resorption and loss (48). Additionally, IL-7 is associated with the number of T lymphocytes. Studies have confirmed that the absence of estrogen after OVX resulted in elevated IL-7 levels, which stimulated thymus-dependent differentiation of bone marrow-derived progenitor cells and thymus-independent peripheral expansion of mature T cells, thereby upregulating T lymphocyte development and inducing bone loss (174). IL-7 secreted by osteoblasts is identified as a key cytokine for B lymphocyte differentiation, in addition to T lymphocytes (175), and is regulated in cells that overexpress osterix via the mechanistic target of rapamycin complex 1 (mTORC1) pathway (176, 177). Bone loss was caused by the IL-7-induced proliferation of B lymphocytes in wild-type rodents, while bone trabeculae and bone volume were substantially increased in IL-7R-deficient mice, indicating that the IL-7-induced increase in B lymphocyte production was also associated with bone resorption (49). Recent research suggests that IL-7/IL-7R may regulate the specific mechanisms of CTSK, NFATc1, and MMP9, as well as the phosphorylation of p38 and Akt, by activating the c-Fos/c-Jun pathway, thereby increasing the number of osteoclasts and amount of bone resorption in RANKL-stimulated macrophages (178). On the other hand, IL-7 can also directly stimulate osteoclast formation by activating STAT5 *via* a pathway independent of RANKL (50).

Nonetheless, other studies on IL-7 suggested a mechanism contrary to the above, and IL-7 may be a potential osteoclast formation inhibitor *in vitro*. One study found that IL-7 inhibited osteoclast formation in bone marrow cells from mice co-cultured with CSF-1 and RANKL (179), and another study found that IL-7-deficient mice had a significant increase in the number of osteoclasts and a significant decrease in bone trabecular bone mass compared with wild-type controls (180). The specific causes for the divergent conclusions should be investigated further.

### Interleukin-8

IL-8 is a cytokine produced by osteoblasts that promotes RANKL-induced osteoclastogenesis in an autocrine manner, which can be inhibited by anti-IL-8 antibodies or IL-8 receptor inhibitors *in vitro* (51). Although human osteoclasts produce high levels of IL-8, the research on IL-8 is still lacking. One of the possible reasons is the lack of IL-8 equivalent in rodents, which makes it difficult to model human diseases. Therefore, finding an effective IL-8 equivalent in rodents may be the direction for future IL-8 research.

### Interleukin-10

IL-10 is a type of Th2 cytokine. The levels of IL-10 were substantially lower in osteoporosis patients than in healthy individuals (181). However, IL-10 levels increased in osteoporosis patients following anti-osteoporosis treatment (182). Animal experiments in which the IL-10 expression was significantly reduced in an osteoporosis model of postmenopausal mice (183) and IL-10-deficient mice exhibited decreased bone mass, increased mechanical fragility, and inhibited bone formation (184), indicating a correlation between IL-10 levels and the development of osteoporosis.

IL-10 is produced by activated T and B lymphocytes (185) and is a direct inhibitor of both osteoclast and osteoblast formation through the RANK/RANKL/OPG axis.

IL-10 inhibits the early phases of osteoclast progenitor cell differentiation into osteoclast precursors during osteoclast differentiation (52). Specifically, IL-10 inhibits osteoclast differentiation by increasing OPG to inhibit RANKL expression and by decreasing RNAK and M-CSF expression (53). IL-10 inhibits RANK-induced osteoclast formation and inhibits calcium signaling, downstream of RANK, via TREM-2 transcriptional inhibition (54). IL-10 also inhibits NFATc1 expression and nuclear translocation by inhibiting c-Fos and c-Jun, thereby inhibiting osteoclastogenesis (55). In OVX mice with osteoporosis, the number of regulatory B (B10) cells that produce IL-10 decreased and the number of IL-17-producing Th17 cells increased compared with control mice. However, the transplantation of B10 cells reduced the number of Th17 cells and inhibited the development of osteoporosis (186), suggesting that B10 cell therapy for osteoporosis may be feasible.

For osteogenic differentiation, IL-10 inhibits bone marrow osteogenic activity by preventing bone mineralization in mouse bone marrow cells and inhibiting the synthesis of bone proteins, including alkaline phosphatase (ALP), type I collagen, and osteocalcin (56).

### Interleukin-12

IL-12 is an anti-osteoclast factor that inhibits RANKL-induced osteoclastogenesis through the suppression of NFATc1 (61) or the promotion of osteoblast apoptosis via the Fas/FasL pathway (62). IL-12 also has a synergistic effect with IL-18 in the mechanism of osteoclast apoptosis induction, as will be discussed in the following section. There are not many studies on IL-12 in osteoporosis, and further exploration is still needed.

### Interleukin-13

IL-13 is a Th2 anti-osteoclast cytokine which is analogous to IL-4. IL-4 and IL-13 share a specific endothelial cell surface IL-4/IL-13-receptor complex so they have common biological effects and similar downstream intracellular signaling pathways. IL-13 inhibits osteoclast formation by activating STAT6 in endothelial cells to induce OPG expression (36). By inhibiting osteoblast cyclooxygenase-2 (COX-2)-dependent prostaglandin synthesis, IL-13 and IL-4 can also inhibit bone resorption (63).

### Interleukin-15

IL-15, similar to IL-7, is a member of the IL-2 super family and shares many mechanisms of action with IL-2. However, IL-2 cannot be substituted for its function in promoting the differentiation of osteoclast progenitors into osteoclast precursors (64). Additionally, IL-15 also has an indirect stimulatory effect on osteoclast formation, operating synergistically with RANKL to induce osteoclast formation primarily via activating extracellular signal-regulated kinase (ERK) to mediate this synergistic effect (65). In a co-culture environment with mouse bone marrow cells and osteoblasts, IL-15 treatment also increased caspase3 expression in NK cells in a dose-dependent manner, thereby promoting osteoblast apoptosis (66).

### Interleukin-17/interleukin-25

Interleukin-17 (IL-17), a cytokine secreted by Th17 cells, is closely associated with osteoclastic effects given that Th17 cells represent a significant subpopulation of osteoclasts. In the bone marrow cells of estrogen-deficient OVX mice, both the number of Th17 cells and the level of circulating IL-17 were elevated, and increased IL-17 levels promoted the expression of pro-osteoclastic cytokines such as TNF-α, IL-6, and RNAKL in osteoblasts, thereby inducing bone loss (67). Estrogen or anti-IL-17 treatments can alleviate the symptoms of bone loss (67, 68). Administration of an anti-IL-17 neutralizing antibody can promote the regeneration of new bone in osteoporotic fractures by enhancing the activity of FOXO1 and ATF4, enhancing the expression of osteogenic markers, and decreasing the oxidative stress in the injured fraction (69). The fact that Th17 cells and IL-17 levels are associated with bone loss has also been verified in humans, where both Th17 cell frequency and IL-17 levels were higher in postmenopausal women than in premenopausal women (70). The result also correlates with reduced bone mineral density (70). IL-17 not only induces osteoclasts by elevating the expression of osteoclastic cytokines RANKL (71), TNF-α, IL-1, IL-6, and IL-8 (72, 73), but also promotes autophagy of osteoclast precursors and osteoclast formation via JNK signaling in a low dose-dependent manner (74).

However, it appears that the function of IL-17 changes with increasing concentrations. Other studies have demonstrated that high concentrations of IL-17 inhibited the differentiation of osteoclast precursors into osteoclasts (75). High concentrations of IL-17 inhibited matrix protein hydrolysis during bone resorption by downregulating the expression of histone K and MMP-9 in osteoclasts (75). In addition, IL-17 can induce proliferation, migration, motility, and osteoblast differentiation of human bone marrow-derived mesenchymal stem cells (hMSCs) in a manner dependent on ROS and MEK/ERK (76). At the same time, IL-17 induces the expression of M-CSF and RANKL on hMSCs to support the *in vitro* and *in vivo* osteoclast formation process (76).

In addition to the well-researched effects on the osteoclastic process, the role of IL-17 in osteoblast differentiation has also been examined. IL-17 has a positive effect on the early differentiation of primary osteoblasts, as well as an inhibitory effect on osteoblast calcification (77), and it inhibits osteoblast differentiation and bone regeneration processes in rodents (78). *In vitro*, high concentrations of IL-17 also induce osteoblast searing via the NLRP3 inflammatory vesicle pathway, triggering the release of IL-1 and RANKL and disrupting bone metabolism even further (79).

### Interleukin-18

IL-18, also known as interferon-gamma-inducing factor (IGIF), is a pleiotropic pro-inflammatory cytokine with similar functions to IL-12 (187). In the case of TNF-α-induced Fas, analogous to IL-12, IL-18 can inhibit TNF-α-induced osteoclastogenesis by mediating myeloid apoptosis via Fas/FasL (80, 81). In contrast, another study demonstrated that anti-FasL antibodies were unable to completely inhibit apoptosis induced by the pathway described above (82). In the presence of TNF-α, IL-12 and IL-18 induce nitric oxide (NO) production in a synergistic manner, which also leads to apoptosis (82). Additionally, IL-18 induces the production of IFN-γ and GM-CSF in T cells, inhibiting osteoclast formation indirectly (83, 84).

IL-18 binding protein (IL-18BP) is an antagonist of IL-18 with anti-inflammatory properties. The treatment of OVX mice with IL-18BP prevented bone loss, and in women with osteoporosis, IL-18BP levels decreased while serum IL-18 levels increased (188), suggesting that IL-18BP may be used to treat postmenopausal osteoporosis.

### Interleukin-19

Interleukin-19 (IL-19) is an inhibiting cytokine that belongs to the IL-10 family. By inhibiting NF-κB and p38MAPK activation and c-Fos expression, IL-19 inhibits RANKL-induced osteoclast differentiation. IL-19 also maintains the osteoclast precursor state, including monocytes and macrophages in an autocrine manner (85). In addition, IL-19 can promote the release of other pro-inflammatory cytokines such as TNF-α, IL-1β, and IL-6 while upregulating RANKL expression in synovial fibroblasts, which further promote the osteoclasts differentiation in arthritis (189). However, the research on IL-19 in osteoporosis is still needed to find more cytokines interactions and therapeutic targets.

### Interleukin-20

IL-20, a member of the IL-10 family, has been shown to have higher serum concentrations in osteoporotic patients than in healthy control patients, and to be substantially upregulated in the serum of OVX mice (86). Anti-IL-20 monoclonal antibody treatments can inhibit M-CSF and RANKL-induced osteoclast differentiation *in vitro* (86) and may represent a potential therapy for preventing osteoporotic bone loss. In particular, IL-20 induces the expression of RANK in M-CSF-derived osteoclast precursors and promotes the transduction of osteoclastic signals such as NF-κB, TRAF6, STAT3, NFATc1, and c-Fos (86). IL-20 also induces the expression of cathepsin G in osteoclasts, thereby increasing the level of soluble RANKL (86). IL-20 can also upregulate RANKL expression in osteoblasts via an autocrine mechanism (86). Another study demonstrated that IL-20 inhibited the survival and differentiation of osteoblasts by upregulating sclerostin and downregulating osterix, RUNX2, and OPG (87).

### Interleukin-23

IL-23, a member of the IL-6/IL-12 family, can participate in T-cell-mediated osteoclast formation by inducing the differentiation of naive CD4(+) T cells into Th17 cells, which secrete IL-17 for further action (88). Therefore, it is called the IL-23/IL-17 axis. In a lipopolysaccharide-induced model of inflammatory bone destruction, mice deficient in IL-17 or IL-23 exhibited significantly less bone loss (88). In addition, IL-23 has a pathway independent of IL-17 that modulates osteoclast differentiation by upregulating RANK expression in bone marrow precursor cells (89) and RANKL expression in CD4(+) T cells (90). However, the role of IL-23 on bone *in vivo* is also controversial, as one study discovered that IL-23 indirectly inhibited osteoclast formation *in vitro* in a CD4(+) T lymphocyte-dependent and dose-dependent manner (91). IL-23 can also increase bone mass in long bones by limiting resorption of immature bone formation below the growth plate (91).

### Interleukin-27

IL-27, also a member of the IL-6/IL-12 family, functions via the IL-27R (IL-27R/WSX-1) and gp130 complexes. IL-27 is also a potent anti-osteoclastogenic factor that inhibits RANK downstream MAPK and NF-κB signaling pathways to eliminate RANKL-induced c-Jun and NFATc1 expressions (92). In osteoclast precursors, IL-27 downregulates TREM-2 co-stimulatory receptor expression and thereby inhibits NFATc1 action (92). In addition, IL-27 can inhibit osteoclast formation by STAT1-dependently downregulating the transcription factor c-Fos (93). In addition, another study discovered that IL-27 inhibited the secretion of RANKL and sRANKL via STAT3 in the surface of CD4(+) T cells (94). In recent years, a new study determined that IL-27 affected both osteoblasts and osteoclasts through early growth response-2 (Egr-2) and that IL-27 treatment in OVX mice led to the loss of bone trabecular structures and the preservation of cortical bone parameters (95). The reason for this is that IL-27 inhibits the differentiation of Th17 cells via the suppression of the transcription factor RORγt, activates Egr to induce IL-10-producing Tr1 cells, and inhibits osteoblast apoptosis by inducing anti-apoptotic factors such as MCL-1 via Egr-2 (95). IL-27 also inhibits osteoclastogenesis in an Egr2-dependent mechanism. It upregulates the expression of the RNAKL repressor Id2, which was also demonstrated in female patients with osteoporosis whose serum IL-27 levels were reduced along with decreased Egr2 expression (95), suggesting a potential new anti-osteoporosis treatment strategy.

### Interleukin-29

IL-29, also known as interferonλ1 (IFNλ1), is a member of the IFN family along with IL-28A and IL-28B, which shares the same receptor complex (IL-28R1/IL-10R2), activates the downstream JAK-STAT signaling pathway upon binding, and transcribes numerous IFN-related genes (190). *In vitro* and *in vivo*, IL-29 derived from dendritic cells inhibits osteoclast formation and bone resorption activity (96). IL-29 inhibits RANKL-induced osteoclast formation by activating the STAT signaling pathway, blocking NF-κB activation and NFATc1 translocation, and repressing osteoclast gene expression (97).

### Interleukin-32

IL-32 belongs to a class of inflammatory cytokines that elicits a variety of other cytokines, of which IL-32γ is one isoform. Age-related increases in bone formation and osteogenic capacity were observed in mice that overexpress IL-32γ (98). They were protected from OVX-induced osteoporosis more than wild-type mice, which may be mediated by the upregulation of miR-29a (98). There is also a correlation between reduced plasma IL-32γ levels and BMD in humans (98), suggesting a protective mechanism for IL-32 against bone loss.

### Interleukin-33

IL-33, a member of the IL-1 family with the specific receptor orphan IL-1 receptor ST2 (IL-1R-like 1), is an osteoprotective factor that inhibits osteoclast formation by at least three mechanisms. Postmenopausal women with osteoporosis had substantially lower IL-33 levels than healthy control women (191). IL-33 interacts with its specific receptor ST2 and inhibits RANKL-induced NFATc1 expressions and nuclear translocations by regulating the expression of Blimp-1 and interferon regulatory factor-8 (IRF-8), thereby suppressing RANKL-induced osteoclast formation and osteoblast-related gene expression (99, 100). According to another study, IL-33 induced osteoclasts apoptosis by increasing the expression of pro-apoptotic molecules such as Bcl-2-associated X protein (BAX), Fas, FasL, and Fas-associated death structural domains (101). In the bone marrow culture state, IL-33 induces mRNA expression of GM-CSF, IL-4, IL-13, and IL-10 to inhibit osteoclast formation (192). The IL-33/ST2 signaling pathway on the aforementioned anti-osteoclast production, which is also associated with vitamin D (100). IL-33 inhibited TNF-induced osteoclast formation and bone resorption, as shown in a mouse model in which mice that were overexpressing TNF-α and treated with IL-33 exhibited a significant reduction in bone loss (102). Osteoprotective IL-33 induces osteoclast precursors to differentiate into CD206(+) alternatively activated macrophages (AAM) rather than osteoclasts in an autocrine manner via GM-CSF (102). In another study, mice lacking the ST2 receptor displayed typical bone formation, but increased bone resorption and decreased bone trabecular bone mass (193). Additionally, IL-33 stimulates osteoblast function, promotes matrix mineral deposition, and reduces sclerostin mRNA levels in primary osteoblasts treated with ascorbate for an extended period of time (103).

### Interleukin-34

M-CSF, an essential cytokine for osteoclast formation, binds to the receptor c-Fms (CSF-1R) to exert its biological effects. Interleukin-34 (IL-34), a second ligand that can bind to c-Fms, can replace the function of M-CSF and is another crucial factor in osteoclast formation. In combination with RANKL, it can induce osteoclast differentiation and bone resorption, and systemic administration of IL-34 to mice decreased trabecular bone mass (104). Another study found that IL-34 promoted the proliferation and differentiation of bone marrow macrophages toward osteoclasts by increasing the expression of NFATc1, stimulating the expression of p-STAT3, and inhibiting the expression of Smad7 without M-CSF (105). In addition, a recent study discovered that low-dose IL-34 regulated hBMSCs osteogenesis and enhanced fracture healing in part via the PIK/AKT and ERK signaling pathways but had no effect on osteoclast formation *in vitro* or osteoporosis *in vivo* (106).

### Interleukin-35

IL-35, belonging to the IL-12 family, is a novel class of anti-inflammatory and immunosuppressive factors. In bone immunology, IL-35 is a direct inhibitor of osteoclast formation, preventing TNF-induced osteoclast formation and bone resorption *in vitro* and inhibiting osteolysis *in vivo* (107). Specifically, IL-35 inhibits NFATc1, c-Fos, and TRAP through the NF-κB and MAPK pathways, which also inhibits TNF-induced osteoclast formation and promotes apoptosis through JAK1/STAT1 activation (107).

Another imbalance in osteoporosis is the imbalance between bone and adipogenesis (194). MSCs have the ability to differentiate into osteoblasts and adipocytes at the same time, which is affected by cytokines and hormone regulation in the microenvironment (195). IL-35 stimulates the proliferation of MSCs while inhibiting their apoptosis or differentiation toward adipogenic. Specifically, it increases the expression of β-catenin and Axin2, which are essential factors in the differentiation of MSCs into osteoblasts in Wnt/β-catenin-PPARγ pathway (108). IL-35 controls the equilibrium between osteogenic and lipogenic differentiation of progenitor cells, suggesting its potential application in osteoporosis and obesity intervention.

IL-35 can also be involved in RANKL and M-CSF-induced osteoclasts and angiogenesis via the Th17/IL-17 axis, exhibiting inhibitory effects in both metabolic processes commonly associated with osteoporosis (196), thereby indicating a potential therapeutic direction.

However, it has also been discovered that IL-33 stimulated the phosphorylation of relevant signaling molecules such as Syk, phospholipase Cγ2, Gab2, MAP kinases, TAK-1, and NF-κB in human CD14(+) monocytes via ST2, thereby promoting functional osteoclast formation, and also increased the expression of various osteoclast differentiation factors including TRAF6, NFATc1, c-Fos, C-Src, histone K and calcitonin receptor, and ultimately also induced bone resorption (109).

### Interleukin-37

IL-37, a member of the IL-1 family, has received relatively less attention than the above cytokines. Nonetheless, its roles in inhibiting osteoclast activity and in bone resorption have been identified. IL-37 inhibits osteoclast formation and pathological bone resorption induced by lipopolysaccharide (110). IL-37 also plays a significant function in the osteogenic differentiation of MSCs, significantly increasing the expression of osteoblast specific genes to accelerate bone healing in a rat skull defect model via PI3K/AKT activation, but the specific mechanism needs further research (111).

## Interferon

IFN are categorized as IFN-α, IFN-β, and IFN-γ, and each has a distinct function in bone remodeling.

IFN-α and IFN-β have inhibitory effects on RANKL-induced osteoclast formations, which can inhibit RANKL-induced osteoclastogenesis by reducing c-Fos (112, 113). A study also showed that IFN-β inhibited osteoclastogenesis by increasing NO production and the inducible nitric oxide synthase (iNOS) signaling pathway (114).

IFN-γ acts on both osteoblasts and osteoclasts (197), with osteoblasts producing modest levels of IFN-γ to stimulate the expression of osteoblast differentiation genes such as Runx2, osterix, ALP, and osteocalcin, further leading to osteoblast differentiation (115, 116). The knockdown of the IFN-γ receptor simultaneously inhibits this effect (117). IFN-γ can downregulate c-fms expression and thus counteract the effect of M-CSF on osteoclast precursors (118), as well as inhibit NFATc1 expression by stimulating TRAF6 degradation through the ubiquitin/proteasome system, which in turn inhibits downstream JNK and NF-κB (8, 197). IFN-γ can also mediate osteoclast apoptosis through the Fas/FasL signaling pathway (119). The above three pathways can inhibit the differentiation and function of osteoclast. IFN-γ also has a positive effect on osteoclastogenesis in late differentiation. It can stimulate osteoclast fusion by the expression of dendritic cell-specific transmembrane protein (DC-STAMP) through the upregulation of NFATc1 and c-Fos (120) and can promote osteoclastogenesis by CXCL10/Interferon-gamma induced protein 10 (IP-10) secretion by macrophages to stimulate RANKL and TNF-α secretion by T cells (121).

## Chemokine

Numerous studies have demonstrated that chemokines influence the differentiation and function of osteoblasts and osteoclasts, regulating bone formation and resorption via autocrine or paracrine mechanisms. The current status of research on the role of various chemokines in bone remodeling has been systematically reported in this article (198). Among these chemokines, CCL2, CCL3, and CCL20 are the most researched types and we will focus on these three chemokines. CCL2 and CCL3 function as pro-osteoclastic cytokines, stimulating the osteoclastogenesis (198). In osteoporosis, CCL2 binding to its receptor C-C chemokine receptor-2 (CCR2) and then activates NF-κB and ERK1/2 signaling, which further lead to increase of RANK expression and RANKL-induced osteoclastogenesis (199). Patients with postmenopausal osteoporosis showed a significant increase in serum CCL3 compared with other groups, indicating that CCL3 may be a potential biomarker to predict disease severity of postmenopausal osteoporosis (200). CCL20 can not only stimulate the osteoclastogenesis but can also act on osteoblast differentiation (198). Other chemokines are not described in detail here.

## Conclusion

**Figure 2:**
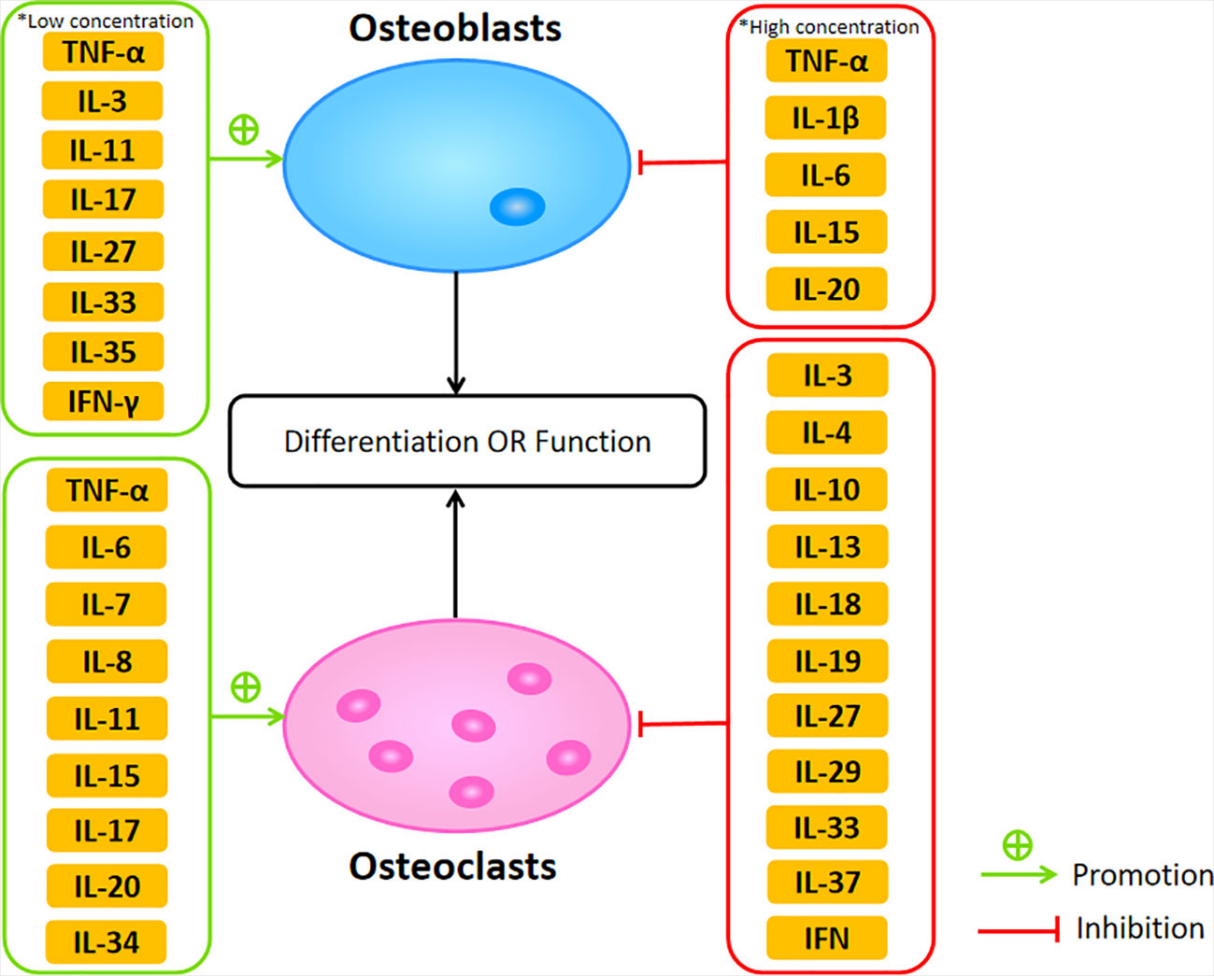
The promotive and suppressive effects of different cytokines on osteoblasts and osteoclasts affecting their differentiation or function.

The cytokines network plays a critical role in maintaining the balance of bone resorption and formation between osteoclasts and osteoblasts. Dysregulation of cytokines may result in bone diseases such as osteoporosis. Among these cytokines, TNF-α, IL-1, IL-6, IL-7, IL-8, IL-11, IL-15, IL-17, and IL-20 belong to osteoclastogenic cytokines, mainly promoting osteoclastogenesis. Meanwhile, anti-inflammatory cytokines, including IL-3, IL-4, IL-10, IL-13, IL-18, IL-19, IL-27, IL-29, IL-32, IL-33, IL-37, and IFN, function as anti-osteoclastogenic cytokines inhibiting the process of osteoclastogenesis (**Figure 2**). Although there has been a great deal of research focus on the effect of proinflammatory cytokines in the bone remodeling of osteoporosis, the cross talk between bone and immune system remains complex, leading to the difficulty of transformation into clinical practice. However, owing to the effort made by many scientists, some molecular-targeted drugs are in clinical trials and have achieved certain results. For example, benzydamine, a non-steroidal anti-inflammatory drug, can inhibit osteoclast differentiation and bone resorption through downregulating the expression of IL-1β (201). Oral administration of lactulose could downregulate pro-osteoclastogenic cytokines levels including TNF-α, IL-6, RANKL, and IL-17 as well as upregulate the anti-inflammatory cytokine IL-10 in OVX mice, which ameliorated estrogen deficiency-induced bone loss in these mice (202). In this review, we discussed the concrete effects of various cytokines in osteoblasts and osteoclasts during bone remodeling in osteoporosis, summarizing the current research and providing multiple therapeutic targets for further study. Therefore, we hope our review is also helpful in the development of osteoporosis research and treatment.

## Author contributions

JX and LY drafted and wrote the manuscript. LW revised the manuscript. FL reviewed and edited the manuscript. ZD assisted in reviewing materials and organizing manuscripts. All authors contributed to the article and approved the submitted version.
